# Supplementary material for: The PANDEMYC Score. An Easily Applicable and Interpretable Model for Predicting Mortality Associated With COVID-19
Source: J Clin Med. 2020 Sep 23;9(10):3066. doi: 10.3390/jcm9103066 (PMC7598151; doi:10.3390/jcm9103066)
Supplement: Supplementary file 1 [file jcm-09-03066-s001.pdf]

SUPPLEMENTARY MATERIAL

Supplementary Table S1. Results obtained with different models in the training, validation, and test tables, ordered from the highest to lowest AUC in the test table.

Supplementary Table S2 Demographics and comorbidity data for 1,968 patients hospitalized with COVID-19 stratified into 3 samples (training, validation, and test).

Supplementary Table S3. TRIPOD Checklist: Prediction Model Development and Validation

Supplementary Figure S1. Probability calibration according to test data.

Supplementary Table S4. Comparisons between the different models based on the AUC values. The DeLong method was used for the comparisons, and a Bonferroni correction was applied for the multiple comparisons.

Supplementary Table S1. Results obtained with different models in the training, validation, and test tables, ordered from the highest to lowest AUC in the test table.

Evaluation of the predictive capacity of the model by comparison with other competitive machine learning methods

Model	AUC Training	AUC Validation	AUC Test
Gradient Boosting	0.975	0.836	0.884
WoE Methodology	0.865	0.808	0.883
Random Forest	1	0.832	0.882
SVM Linear Kernel	0.869	0.812	0.869
Neural Network	0.929	0.792	0.852
SVM Quadratic Kernel	0.921	0.736	0.818

Evaluation of the predictive capacity of the model by comparison with other competitive machine learning methods. We used 3 tables to apply the WoE methodology: one for training, one for validation and one for testing and selected, respectively, 60% (1182), 20% (393), and 20% (393) of the patients at random. The role played by these tables in each of the steps of this methodology is as follows:

- **Generation of WoE variables** - The trees used to generate these variables are generated from the training table and pruned with the validation table. Pruning is based on evaluation of whether the proportions of deaths observed in the training table in the different leaves of the tree correspond to those observed in a different sample, the validation table. If this is not the case, the leaf is pruned. This pruning process seeks to guarantee the generalization capacity of the WoE-type variables.

- **Logistic regression model fitting** - Given the small number of observations available for model fitting, training and validation samples have been used together during the stepwise selection process of WoE-type variables by applying a cross-validation strategy.

We compared our model with competitive machine learning techniques. These techniques are listed below, along with a description of the configuration obtained with the best model obtained for each of them:

- **Neural Network (NN)** - we used a perception with a single hidden layer in which the number of neurons had been established according to Bishop's rule (log2(number of patterns in the training table) = log2(1182)).

- **Supported Vector Machine (SVM)** - one model with linear kernel function (SVM1) and another with quadratic kernel function (SVM2) was adjusted.

- **Random Forest (RF) –** 1,000 trees with a maximum depth of 50 were combined.

- **Gradient Boosting (GB) –** 1,000 trees with a maximum depth of 3 were combined. The shrinkage parameter took the value of 0.001.

Supplementary Table S2. Demographics and comorbidity data for 1968 patients hospitalized with COVID-19 stratified into 3 samples (training, validation, and test).

Characteristic	All data N=1968	Training n=1181	Validation n=393	Test n=394	P value	P value	P value
**Male sex, No. (%)**	1,104 (56.4)	651 (55.4)	227 (58.4)	226 (57.5)	0.393	0.474	0.967
**Age, median (IQR)**	67 (27)	67 (28)	67 (25)	66 (24)	0.999	0.999	0.999
**Born in Spain, No. (%)**	1,413 (75.6)	846 (75.2)	292 (77.9)	275 (74.3)	0.338	0.527	0.184
**Dead, No. (%)**	325 (16.5)	195 (16.5)	64 (16.3)	66 (16.8)	0.979	0.974	0.936
**Comorbid conditions, No. (%)**							
**Chronic heart disease**	415 (21.7)	250 (21.7)	83 (21.9)	82 (21.5)	0.999	0.937	0.985
**Hypertension**	991 (51.8)	610 (53)	193 (50.8)	188 (49)	0.415	0.195	0.749
**Chronic pulmonary disease**	238 (12.5)	128 (11.2)	53 (14)	57 (15)	0.182	0.065	0.769
**Asthma**	156 (8.2)	92 (8)	27 (7.1)	37 (9.7)	0.626	0.37	0.245
**Stage 4 chronic kidney disease**	110 (5.8)	74 (6.4)	19 (5)	17 (4.5)	0.358	0.189	0.858
**Liver cirrhosis**	31 (1.6)	20 (1.7)	6 (1.6)	5 (1.3)	0.999	0.726	0.997
**Solid neoplasm (active)**	81 (4.2)	38 (3.3)	21 (5.5)	22 (5.7)	0.077	0.049	0.999
**Hematologic neoplasm (active)**	34 (1.7)	21 (1.8)	6 (1.5)	7 (1.8)	0.914	0.999	0.999
**HIV infection**	11 (0.6)	6 (0.5)	1 (0.3)	4 (1)	0.828	0.465	0.371
**Obesity**	284 (17.7)	169 (17.6)	62 (19.3)	53 (16.6)	0.529	0.734	0.411
**Diabetes**	436 (22.9)	250 (21.9)	90 (23.7)	96 (25.1)	0.514	0.209	0.689
**Dyslipidemia**	444 (36.8)	263 (36.4)	99 (39.3)	82 (35)	0.261	0.593	0.169
**Inflammatory disease**	99 (5.2)	57 (5)	20 (5.3)	22 (5.7)	0.941	0.643	0.881
**Dementia**	108 (5.7)	72 (6.3)	17 (4.5)	19 (5)	0.234	0.416	0.871
**Malnutrition**	40 (2.3)	30 (2.9)	3 (0.9)	7 (2)	0.054	0.500	0.342
**Smoking**	460 (28)	265 (27.2)	109 (32.4)	86 (25.8)	0.039	0.855	0.066
**Current medications, No. (%)**							
**Nonsteroidal anti-inflammatory drugs**	52 (3.2)	30 (3.1)	13 (4)	9 (2.7)	0.529	0.924	0.513
**Angiotensin-converting enzyme inhibitors**	432 (23.4)	258 (23.4)	91 (24.5)	83 (22.4)	0.638	0.799	0.535
**Angiotensin II receptor blockers**	293 (15.9)	193 (17.6)	51 (13.8)	49 (13.2)	0.129	0.075	0.904
**Inhaled corticosteroids**	173 (9.4)	95 (8.6)	37 (10)	41 (11)	0.457	0.18	0.729
**Systemic corticosteroids**	55 (3)	32 (2.9)	9 (2.4)	14 (3.8)	0.788	0.491	0.401
**Vital signs at admission, median (IQR)**							
**Temperature, ºC**	37 (1.3)	37 (1.2)	37 (1.5)	37 (1.3)	0.999	0.999	0.999
**Heart rate, bpm**	89 (21)	89 (23)	89 (19.75)	89 (22)	0.867	0.867	0.782
**Oxygen saturation in room air %**	94 (7)	94 (6)	94 (6)	95 (6.75)	0.669	0.669	0.669
**Admission signs and symptoms, No. (%)**							
**Fever**	1,422 (75.1)	855 (75.3)	269 (71.2)	298 (78.4)	0.151	0.234	0.03
**Malaise**	792 (42.3)	466 (41.7)	159 (42.2)	167 (44.3)	0.771	0.334	0.634
**Upper respiratory tract symptoms**	421 (22.4)	256 (22.7)	88 (23.5)	77 (20.4)	0.821	0.408	0.371
**Dyspnea**	1,071 (56.8)	648 (57.4)	197 (52)	226 (59.5)	0.115	0.422	0.05

**Chest pain**	183 (9.7)	124 (11)	23 (6.1)	36 (9.4)	0.008	0.497	0.106
**Cough**	1,258 (66.7)	758 (67.2)	240 (63.3)	260 (68.4)	0.294	0.556	0.174
**Sputum production**	232 (12.3)	134 (11.9)	44 (11.7)	54 (14.3)	0.999	0.246	0.338
**Hemoptysis**	33 (1.8)	20 (1.8)	7 (1.9)	6 (1.6)	0.999	0.998	0.996
**Myalgia/arthralgia**	374 (20)	207 (18.4)	73 (19.6)	94 (24.9)	0.693	0.007	0.084
**Headache**	180 (9.6)	109 (9.7)	34 (9.1)	37 (9.8)	0.807	0.999	0.812
**Altered consciousness**	95 (5)	69 (6.1)	14 (3.7)	12 (3.2)	0.105	0.041	0.837
**Seizures**	8 (0.4)	6 (0.5)	1 (0.3)	1 (0.3)	0.828	0.826	0.999
**Abdominal pain**	65 (3.5)	44 (3.9)	12 (3.2)	9 (2.4)	0.641	0.225	0.654
**Vomiting/nausea**	219 (11.6)	138 (12.2)	44 (11.7)	37 (9.8)	0.864	0.245	0.474
**Diarrhea**	327 (17.3)	196 (17.4)	68 (18)	63 (16.6)	0.805	0.839	0.69
**Skin rash**	10 (0.5)	5 (0.4)	3 (0.8)	2 (0.5)	0.681	0.999	0.998
**Laboratory findings, median (IQR)**							
**Hemoglobin - g/L**	13.8 (2.2)	13.8 (2.125)	13.8 (2.3)	13.7 (2.1)	0.976	0.976	0.913
**WBC count x10⁹/L**	6,630 (3870)	6,800 (3980)	6,470 (3530)	6,350 (3,690)	0.179	0.179	0.953
**Lymphocyte count - cells/μL**	1,000 (600)	1,000 (600)	1,000 (675)	1,000 (600)	0.4777	0.832	0.710
**Neutrophil count - cells/μL**	4,900 (3,500)	5,050 (3,600)	4,900 (3400)	4,600 (3,200)	0.161	0.042	0.587
**Hematocrit %**	41.2 (6.4)	41.2 (6.4)	41.5 (6.125)	41.2 (6)	0.721	0.782	0.721
**Platelets x10⁹/L**	209 (109)	209 (109)	210 (119.5)	208 (103)	0.999	0.999	0.999
**APTT**	26.1 (4)	26.1 (3.9)	26.3 (4.025)	26.1 (4.1)	0.999	0.999	0.999
**INR**	1.09 (0.15)	1.09 (0.14)	1.09 (0.15)	1.09 (0.15)	0.5926	0.999	0.999
**AST - U/L**	35 (30)	35 (29)	35 (28)	38 (34)	0.999	0.741	0.999
**ALT - U/L**	39 (29)	39 (29.5)	40 (25)	38 (28.75)	0.999	0.999	0.999
**Glucose - mg/dL**	113 (40)	114 (39)	112 (39)	113 (42)	0.999	0.999	0.999
**Creatinine - mg/dL**	1 (0.45)	1.01 (0.4525)	0.985 (0.4625)	0.98 (0.41)	0.999	0.999	0.999
**Sodium - mEq/L**	139 (5)	139 (5)	138 (5)	139 (4.5)	0.999	0.962	0.999
**Potassium - mEq/L**	4.3 (0.7)	4.3 (0.8)	4.3 (0.6)	4.3 (0.6)	0.999	0.999	0.999
**C-reactive protein - mg/L**	67 (104.9)	65.85 (105.875)	64.9 (109.05)	71.7 (101.5)	0.999	0.999	0.999
**Radiology**							
**Pathological chest X-ray on admission, No. (%)**	1711 (91.2)	1025 (90.9)	342 (91.4)	344 (91.7)	0.975	0.859	0.989

Abbreviations: WBC, white blood cell count; APTT, activated partial thromboplastin time; INR, international normalized ratio; ALT, alanine, aminotransferase; AST, aspartate aminotransferase.

Supplementary Table S3. TRIPOD Checklist: Prediction Model Development and Validation

Section/Topic	n		Checklist Item	Page
**Title and abstract**				
Title	1	D;V	Identify the study as developing and/or validating a multivariable prediction model, the target population, and the outcome to be predicted.	1
Abstract	2	D;V	Provide a summary of objectives, study design, setting, participants, sample size, predictors, outcome, statistical analysis, results, and conclusions.	2-3
**Introduction**				
Background and objectives	3a	D;V	Explain the medical context (including whether diagnostic or prognostic) and rationale for developing or validating the multivariable prediction model, including references to existing models.	4
	3b	D;V	Specify the objectives, including whether the study describes the development or validation of the model or both.	5
**Methods**				
Source of data	4a	D;V	Describe the study design or source of data (e.g., randomized trial, cohort, or registry data), separately for the development and validation data sets, if applicable.	6
	4b	D;V	Specify the key study dates, including start of accrual; end of accrual; and, if applicable, end of follow-up.	6
Participants	5a	D;V	Specify key elements of the study setting (e.g., primary care, secondary care, general population) including number and location of centres.	6
	5b	D;V	Describe eligibility criteria for participants.	6
	5c	D;V	Give details of treatments received, if relevant.	6
Outcome	6a	D;V	Clearly define the outcome that is predicted by the prediction model, including how and when assessed.	7
	6b	D;V	Report any actions to blind assessment of the outcome to be predicted.	7
Predictors	7a	D;V	Clearly define all predictors used in developing or validating the multivariable prediction model, including how and when they were measured.	
	7b	D;V	Report any actions to blind assessment of predictors for the outcome and other predictors.	
Sample size	8	D;V	Explain how the study size was arrived at.	7
Missing data	9	D;V	Describe how missing data were handled (e.g., complete-case analysis, single imputation, multiple imputation) with details of any imputation method.	13
Statistical analysis methods	10a	D	Describe how predictors were handled in the analyses.	7
	10b	D	Specify type of model, all model-building procedures (including any predictor selection), and method for internal validation.	7-9
	10c	V	For validation, describe how the predictions were calculated.	7-9

	10d	D;V	Specify all measures used to assess model performance and, if relevant, to compare multiple models.	7-9
	10e	V	Describe any model updating (e.g., recalibration) arising from the validation, if done.	7-9
Risk groups	11	D;V	Provide details on how risk groups were created, if done.	7-9
Development vs. validation	12	V	For validation, identify any differences from the development data in setting, eligibility criteria, outcome, and predictors.	7-9
**Results**				
Participants	13a	D;V	Describe the flow of participants through the study, including the number of participants with and without the outcome and, if applicable, a summary of the follow-up time. A diagram may be helpful.	9
	13b	D;V	Describe the characteristics of the participants (basic demographics, clinical features, available predictors), including the number of participants with missing data for predictors and outcome.	9
	13c	V	For validation, show a comparison with the development data of the distribution of important variables (demographics, predictors and outcome).	9
Model development	14a	D	Specify the number of participants and outcome events in each analysis.	10
	14b	D	If done, report the unadjusted association between each candidate predictor and outcome.	10
Model specification	15a	D	Present the full prediction model to allow predictions for individuals (i.e., all regression coefficients, and model intercept or baseline survival at a given time point).	10
	15b	D	Explain how to the use the prediction model.	10
Model performance	16	D;V	Report performance measures (with CIs) for the prediction model.	10
Model-updating	17	V	If done, report the results from any model updating (i.e., model specification, model performance).	10
**Discussion**				
Limitations	18	D;V	Discuss any limitations of the study (such as nonrepresentative sample, few events per predictor, missing data).	13
Interpretation	19a	V	For validation, discuss the results with reference to performance in the development data, and any other validation data.	13
	19b	D;V	Give an overall interpretation of the results, considering objectives, limitations, results from similar studies, and other relevant evidence.	12-13
Implications	20	D;V	Discuss the potential clinical use of the model and implications for future research.	13
**Other information**				
Supplementary information	21	D;V	Provide information about the availability of supplementary resources, such as study protocol, Web calculator, and data sets.	12

Funding	22	D;V	Give the source of funding and the role of the funders for the present study.	14

*Items relevant only to the development of a prediction model are denoted by D; items relating solely to a validation of a prediction model are denoted by V; and items relating to both are denoted by D;V. We recommend using the TRIPOD Checklist in conjunction with the TRIPOD Explanation and Elaboration document.

Supplementary Figure S1. Probability calibration according to test data.

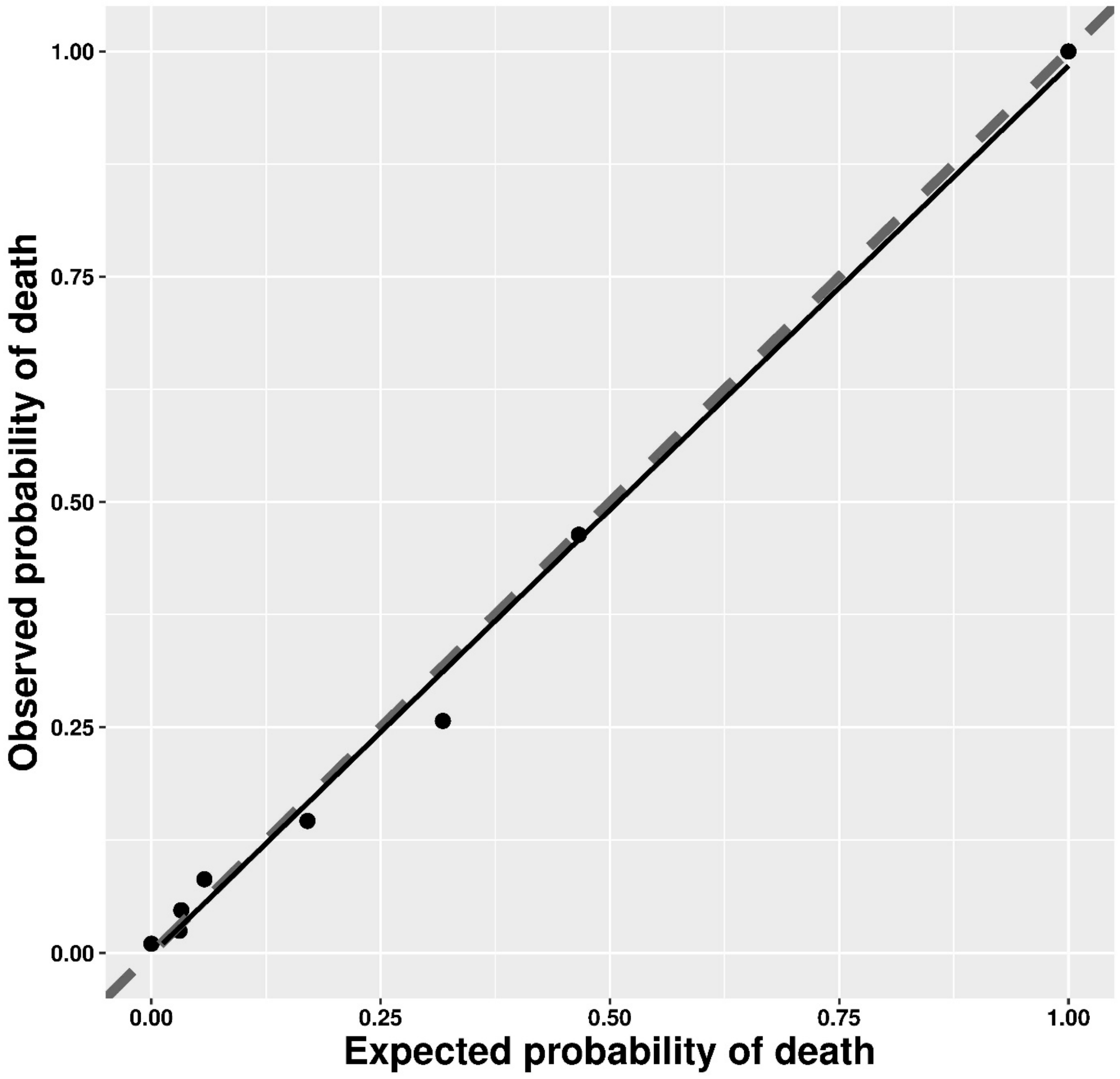

Supplementary Table S4. Comparisons between the different models by AUC value. The DeLong method has been used for the comparisons and a Bonferroni correction applied for the multiple comparisons.

	**NN**	**SVM1**	**SVM2**	**RF**	**GB**
**Logistic**	0.128	0.444	<0.001	0.999	0.999
